# Are Infants Less than 6 Months of Age a Neglected Group for Anemia Prevention in Low-Income Countries?

**DOI:** 10.4269/ajtmh.17-0487

**Published:** 2017-12-18

**Authors:** Cinta Moraleda, Regina N. Rabinovich, Clara Menéndez

**Affiliations:** 1 ISGlobal, Barcelona Centre for International Health Research (CRESIB), Hospital Clínic, Universitat de Barcelona, Barcelona, Spain;; 2 Harvard T.H. Chan School of Public Health, Boston, Massachusetts;; 3 Manhiça Health Research Center (CISM), Manhiça, Mozambique

## Abstract

Anemia is a major public health problem that affects mainly children, predominantly in low-income countries and most often due to iron deficiency (ID). Administration of iron supplements to prevent and treat ID anemia in malaria endemic areas has been controversial for decades; however, recent World Health Organization guidelines recommend universal iron supplementation for children in highly prevalent anemia settings, including those where malaria is endemic. However, infants younger than 6 months of age have been exempted from this recommendation because ID is not considered prevalent at this age and because of assumptions—without evidence—that they are protected from ID through breast milk. To achieve full impact of anemia prevention targeting infants less than 6 months of age who are at highest risk of ID, operational studies that conclusively demonstrate the effectiveness and safety of delivering iron supplements to young infants in settings with a high burden of infectious diseases, including malaria, are needed.

Anemia is a major public health problem that affects over a third of the global population, causing 68 million disability-adjusted life years (nearly 9% of total).^1,2^ Anemia of any etiology increases the risk of child mortality and morbidity, and specifically iron deficiency (ID) anemia has been associated with harmful effects on cognitive and physical development and on the immune function of children.^2–4^ Children less than 5 years of age, especially those living in low- and middle-income countries, have the highest frequency of anemia, and it is the only age group in which anemia prevalence increased from 1990 to 2010.^1,2^ In these countries, the etiology of anemia is often multifactorial, including a combination of nutritional deficiencies, genetic disorders, and infectious diseases, although ID remains the main cause of anemia globally.^1,2^ Prevention and treatment of ID are based on the administration of iron supplements and/or food fortification.^4,5^ However, for decades, iron supplementation in malaria-endemic areas has been controversial because of reports of increased risk of malaria episodes or severe morbidity in individuals receiving iron supplements.^4^ For this reason, previous World Health Organization (WHO) anemia prevention guidelines for children living in malaria-endemic areas recommended targeting only iron-deficient children^6^; this implied screening children for ID, something logistically difficult in resource-limited settings. Moreover, available markers of ID have important limitations in the presence of infectious diseases and inflammation, both conditions highly prevalent in low-income settings.^7^

A recently published Cochrane review on daily iron supplementation to children concluded that “iron supplementation does not adversely affect children living in malaria-endemic areas” and that “routine iron supplementation should not be withheld from children living in countries where malaria is prevalent and malaria management services are available.”^4^ Based on this information, WHO guidelines now recommend universal daily iron supplementation for children from 6 months of age in settings where anemia is highly prevalent for preventing ID and anemia, without having to test for anemia or ID.^5^ This change in the recommendations on anemia prevention is a fundamental public health achievement, and its implementation is expected to have a significant impact in reducing ID and its consequences, mainly in settings where it is most needed. Nevertheless, this policy exempts infants less than 6 months of age mainly on the basis that ID is not a major problem in this age group for physiological reasons and under the assumption that young infants are protected from ID through breastfeeding.^8^ However, this assumption is based on the observation that during the first months of life, a sufficient quantity of iron is available in the breast milk produced by mothers with adequate iron stores themselves, which is uncommon in many low-income settings.^1^ Thus, in these settings, a high proportion of infants are likely to not achieve normal hemoglobin levels after reaching the nadir at 2–3 months of age, when “physiologic anemia of the infant” occurs.^9^

Young infants are protected from ID if the mother had adequate iron stores during pregnancy; the baby was full-term, with normal birthweight; there was delayed umbilical cord clamping; and sufficient iron was provided from exclusive breastfeeding.^8^ Unfortunately, it is rare for all of these conditions to be met and, therefore, most infants in malaria-endemic, and in low-income, countries are at risk of ID during their first 6 months of life.^10,11^ Even in high-income settings, where all the aforementioned criteria are more frequently met, it is considered that the accumulated iron during pregnancy and the amount of iron available in breast milk during the first 6 months of life are not enough to meet the needs of infants. For this reason, the American Academy of Pediatrics recommends that exclusively breastfed term infants should receive 1 mg/kg/day of iron supplementation starting at 4 months of age and continue until appropriate iron-containing complementary foods have been introduced.^12^ Given the higher frequency of factors associated with ID in infants in resource-poor settings, a clear recommendation for preventing anemia with iron supplementation is needed.

Iron supplementation for low-birthweight and preterm infants beginning at 2 months of age was recommended in previous WHO guidelines. However, in the new guidelines, there is no mention regarding this group of infants, so it is not clear if the previous recommendation is maintained. Nevertheless, infants younger than 6 months without these risk factors are still not included in any policy on ID prevention.^5,6^

There is evidence that normal birthweight full-term infants may present anemia prevalences as high as 80% at 3–6 months of age and as high as 90% at 6–9 months of age.^13,14^ Because ID always precedes ID anemia, preventing ID must begin before anemia is manifested to avoid the harmful consequences of both related conditions. Early anemia in life may irreversibly affect the cognitive development and physical growth of infants. Therefore, it is critical to minimize the risks of ID anemia in the first months of life to prevent its consequences when infants are especially susceptible to ID because of their rapid growth.^5,15^

Delayed umbilical cord clamping is the main proposed strategy to prevent ID and the consequent anemia in young infants.^8^ However, although efficacious, in low-income countries, delayed cord clamping is not always easy to implement in busy maternity wards and even less under the conditions of unassisted deliveries that occur in more than 50% of births in sub-Saharan Africa.^16,17^ Improving maternal iron status has been proposed as an intervention to reduce the risk of anemia in infants; however, the relationship between maternal and fetal anemia has been observed in some studies but not in others,^9,15^ suggesting that the maternal–fetal regulation of iron transport is complex and the factors that determine a newborn’s iron stores are not completely understood. Iron supplementation during pregnancy and lactation period, although beneficial for the mothers, has shown little or no effect in preventing anemia in their babies.^18,19^

There have been, to our knowledge, three randomized placebo-controlled trials of iron supplementation in malaria-endemic countries in which the intervention was targeted to children less than 6 months of age. All of them showed that iron supplementation was efficacious in decreasing anemia prevalence and two of them also evaluated safety, showing that the intervention was safe.^10,20,21^ Thus, the administration of prophylactic oral iron supplements (2 mg/kg/day) from 2 to 6 months of age to infants exposed to malaria has been associated with a reduction in the risk of severe anemia during the first year of life without increasing the risks of malaria or other infectious diseases.^10^

The evaluation of the risk of diarrhea associated with prophylactic iron supplements showed a mild increased risk of diarrhea in children but associated with iron plus zinc supplementation.^4^ No serious adverse events, including accidental overdose, were reported in recent reviews or in studies among young infants.^4,10,21–23^

Given the high rate of anemia in the first months of infancy, and the challenges of managing this through alternative methods (i.e., delayed umbilical cord clamping), protecting children during the short period of maximal risk—when iron stores are minimal and the intake of iron is inadequate— can produce long-term health benefits.^24,25^ It may be that iron supplementation at this time maximizes its utilization, with relatively efficient “iron trapping” during the rapid processes of growth and hemoglobin synthesis.^26^ Thus, beginning administration of iron supplements in infancy earlier than what it is currently recommended may have a health benefit by improving iron status and reducing anemia risk.^3^

The implementation challenge is to develop a sustainable and cost-effective strategy to deploy iron supplements to young infants. In most low-income countries, the Expanded Program on Immunization (EPI) represents an established scheme that would be adequate for a cost-effective implementation of this intervention with iron supplements as drops/syrup. Infants start vaccination in the first months of life, and thus implementing iron supplementation via the EPI scheme would likely cover most of the target population. Operational studies that conclusively demonstrate the effectiveness of delivering iron supplements to young infants through the EPI system and that confirm the safety of this intervention in settings with a high infectious disease prevalence, including malaria-endemic areas, are needed to maximize the public health impact of anemia prevention in infants who are most at risk of ID, those younger than 6 months of age. Finally, implementation of additional efficacious but poorly implemented strategies, such as delayed umbilical cord clamping, must be reinforced.

The launch of the new WHO guidelines on anemia prevention is an opportunity to review this gap in the prevention of anemia in young infants and to advance a cost-effective intervention that could be integrated into existing and functioning health systems.

Box 1 **Summary points**

- An important achievement in anemia prevention in children is the new World Health Organization guideline that recommends universal iron supplementation to children where anemia is highly prevalent, including malaria-endemic areas.
- However, infants younger than 6 months of age have been exempted from this policy because iron deficiency is not considered prevalent at this age, which is only true under conditions that are often difficult to meet in low-income settings.
- Studies on the cost-effectiveness and safety of iron supplementation to young infants are needed to realize the full public health impact of anemia prevention.
